# Sugars, Sweet Taste Receptors, and Brain Responses

**DOI:** 10.3390/nu9070653

**Published:** 2017-06-24

**Authors:** Allen A. Lee, Chung Owyang

**Affiliations:** 1 1500 East Medical Center Drive, Division of Gastroenterology, Department of Internal Medicine, Michigan Medicine, University of Michigan, Ann Arbor, MI 48109-5362, USA; allenlee@med.umcih.edu; 2 3912 Taubman Center, SPC 5362, Ann Arbor, MI 48109-5362, USA

**Keywords:** sweet taste receptors, glucose sensing, nutrient sensing, leptin, hypothalamus

## Abstract

Sweet taste receptors are composed of a heterodimer of taste 1 receptor member 2 (T1R2) and taste 1 receptor member 3 (T1R3). Accumulating evidence shows that sweet taste receptors are ubiquitous throughout the body, including in the gastrointestinal tract as well as the hypothalamus. These sweet taste receptors are heavily involved in nutrient sensing, monitoring changes in energy stores, and triggering metabolic and behavioral responses to maintain energy balance. Not surprisingly, these pathways are heavily regulated by external and internal factors. Dysfunction in one or more of these pathways may be important in the pathogenesis of common diseases, such as obesity and type 2 diabetes mellitus.

## 1. Chemosensory Cells in the Tongue

**Figure 1:**
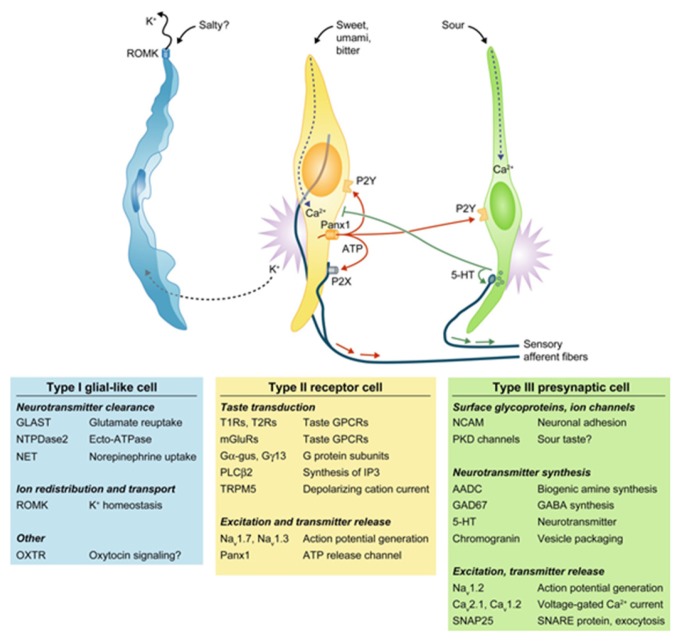
The four major classes of taste cells. This classification incorporates ultrastructural features, patterns of gene expression, and the functions of each Types I, II (receptor), III (presynaptic), and IV (progenitor, not depicted) taste cells. Type I cells (blue) degrade or absorb neurotransmitters. They also may clear extracellular K+ that accumulates after action potentials (shown as bursts) in receptor (yellow) and presynaptic (green) cells. K+ may be extruded through an apical K channel such as renal outer medullary potassium channel (ROMK). Salty taste may be transduced by some Type I cells, but this remains uncertain. Sweet, bitter, and umami taste compounds activate receptor cells, inducing them to release ATP through pannexin 1 (Panx1) hemichannels. The extracellular ATP excites ATP receptors (P2X, P2Y) on sensory nerve fibers and on taste cells. Presynaptic cells, in turn, release serotonin (5-HT), which inhibits receptor cells. Sour stimuli (and carbonation, not depicted) directly activate presynaptic cells. Only presynaptic cells form ultrastructurally identifiably synapses with nerves. Tables below the cells list some of the proteins that are expressed in a cell type-selective manner. AADC, aromatic L-amino acid decarboxylase; Ca, voltage-gated calcium channel; Gα-gus, alpha-gustducin; Gγ13, Gγ13 subunit; GAD, glutamate decarboxylase; GLAST, glutamate aspartate transporter; 5-HT, 5-hydroxytryptamine; mGluRs, metabotropic glutamate receptor; Na, voltage-gated sodium channel; NCAM, neural cell adhesion molecule; NET, norepinephrine transporter; NTPDase; nucleoside triphosphate diphosphohydrolase; OXTR, oxytoxin receptor; Panx1, pannexin 1; PKD, polycystic kidney disease-like channel; PLCβ2, phospholipase C β2; ROMK, renal outer medullary potassium channel; SNAP, synaptosomal-associated protein; T1R, taste 1 receptor family; T2R, taste 2 receptor family; TRPM5, transient receptor potential cation channel M5; Adapted with permission from [6].

Humans can distinguish between five basic tastes, including sweet, salty, umami, bitter, and sour. Recently, lipid sensors have been identified on the tongue which suggests that fat can be considered as the sixth taste [1]. Taste processing is first achieved at the level of taste receptor cells (TRCs) which are clustered in taste buds on the tongue. When TRCs are activated by specific tastants, they transmit information via sensory afferent fibers to specific areas in the brain that are involved in taste perception. Four morphologic subtypes of TRCs have been identified. Type I glial-like cells detect salty taste. Type II cells express G-protein coupled receptors (GPCRs) to detect sweet, umami, and bitter tastes. Type III cells sense sour stimuli, while Type IV cells likely represent stem or progenitor taste cells [2]. Type II cells do not form traditional synapses with afferent nerve fibers. Rather, these cells release ATP through hemichannels, which can then activate purinergic receptors (P2N2 and P2X3) present on the cranial nerve fibers innervating each taste bud (Figure 1) [3,4,5].

Two classes of GPCRs have been identified, including taste 1 receptor family (T1R) and the taste 2 receptor family (T2R) [6]. Two subtypes of the T1R family, including T1R member 2 (T1R2) and T1R member 3 (T1R3), form heterodimers to act as sweet taste receptors [7,8].

## 2. Sweet Taste Signaling

**Figure 2:**
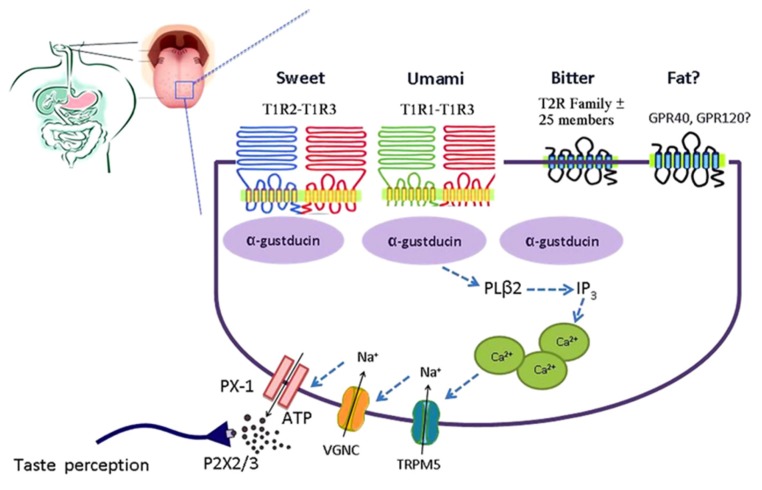
Simplified model of the taste GPCR signaling pathways involved in chemosensing by taste receptors of the tongue. Subtypes of the T1R family heterodimerize to detect sweet (T1R2-T1R3) and umami (T1R1-T1R3). Bitter is detected by the T2R family. Medium-chain and long-chain fatty acids are detected by FFAR1 and GPR120. Taste receptor binding leads to activation of gustatory G-proteins, release of intracellular Ca^2+^, activation of TRPM5, depolarization, activation of voltage-gated Na^+^ channels (VGNC), and release of ATP which activates purinergic receptors on afferent fibers leading to taste perception. ATP, adenosine triphosphate; FFAR1, free fatty acid receptor 1; GPCR, G-protein coupled receptor; PX-1, pannexin 1-hemichannel; T1R, taste receptor type 1; T1R1, taste receptor type 1 member 1; T1R2, taste receptor type 1 member 2; T1R3, taste receptor type 1 member 3; T2R, taste receptor type 2; TRPM5, transient receptor potential cation channel M5; VGNC, voltage-gated Na^+^ channel. Reproduced with permission from [10].

Sweet taste receptors can be activated by a wide range of chemically different compounds, including sugars (glucose, fructose, sucrose, maltose), artificial sweeteners (e.g., saccharin, aspartame, cyclamate), sweet amino acids (d-tryptophan, d-phenylalanine, d-serine), and sweet proteins (monellin, brazzein, thaumatin) [9]. Binding of a ligand to the sweet taste receptor leads to activation of the heterotrimeric G-protein α-gustducin. Phospholipase C β2 is subsequently stimulated, leading to release of intracellular Ca^2+^ and activation of the transient receptor potential cation channel M5 (TRPM5). This sequence results in the release of ATP, which can then activate adjacent sensory afferent neurons that send signals to brain centers involved in taste perception (Figure 2) [10].

Taste cells also express bioactive peptides, including glucagon-like peptide-1 (GLP-1), glucagon, neuropeptide Y, peptide YY (PYY), cholecystokinin (CCK), vasoactive intestinal peptide, and ghrelin [11]. Although the function of these peptides in taste buds is still unknown, their presence suggests a role in the processing and modulation of taste information at the level of the taste bud.

## 3. Chemosensory Cells in the GI Tract

Although taste receptors were initially discovered in taste buds, a growing number of studies have demonstrated that sweet taste receptors are expressed throughout the body, including the nasal epithelium, respiratory system, pancreatic islet cells, and even in sperm and testes [12,13,14].

In the gut, sweet taste receptors are mainly concentrated on enteroendocrine cells. Although these cells represent a small proportion of the total number of epithelial cells in the gastrointestinal (GI) tract, collectively they form the largest endocrine organ in the body [15]. Over 20 different types of enteroendocrine cell types have been identified to date, each of which secretes one or more regulatory peptides or bioactive molecules (Table 1). These hormones can act locally on enteroendocrine cells, on immune cells, nerve endings, or organs at remote sites including pancreatic islets and the central nervous system (CNS). This results in changes in appetite and satiety, inhibition of gastric emptying, stimulation of gastric secretion, pancreatic exocrine and endocrine secretion, induction of nutrient transporters and digestive enzymes, an increase in intestinal barrier function, and modulation of immune responses and tissue growth [15,16,17,18,19].

The function of the sweet taste receptor system in the gastrointestinal tract is likely involved in nutrient sensing, glucose homeostasis, as well as secretion of GI peptides. The intestinal mucosa is highly expressed with taste receptor proteins, including T1R2 and T1R3 [20]. The Na^+^/glucose cotransporter SGLT1 is the major route for transport of dietary sugars from the lumen of the intestine into enterocytes. Dietary sugar and artificial sweeteners increased SGLT1 expression in wild-type mice, but not in T1R3 or α-gustducin knockout mice [21]. Activation of sweet taste receptors on enteroendocrine cells led to increased GLP-1 and glucagon-like insulinotropic peptide (GIP) release, which in turn leads to upregulation of SGLT1 expression. Furthermore, there is a significant decrease in transcript levels of T1R2 following jejunal glucose perfusion in mice [22]. This suggests that sweet taste receptors function as gut luminal nutrient sensors which helps to regulate glucose balance and nutrient intake.

### 3.1. L Cells

Sweet taste receptors are expressed by L cells in the distal small intestine. L cells are distributed throughout the GI tract, with greatest density in the ileum and colon [23]. It has long been known that orally administered glucose triggers a much higher release of insulin compared with intravenous injection of glucose. However, the mechanism was largely unknown until Jang et al. demonstrated that human duodenal L cells express sweet taste receptors that act as glucose sensors in the gut [24]. Activation of L cells by glucose leads to release of hormones, including GLP-1 [25]. GLP-1 leads to increased satiety signals, stimulates insulin release, suppresses glucagon secretion, and slows gastric emptying [26,27]. Glucose-stimulated GLP-1 secretion (GSGS) is severely impaired in *T1R3*-knockout rodents but not in *T1R2*-knockout mice [28]. This suggests that T1R3 can mediate GSGS by itself in the absence of the full sweet taste receptor heterodimer. Furthermore, *SGLT-1*-knockout mice show an 80% reduction in GSGS [29]. These data taken collectively suggest that T1R3 and SGLT1 interact in L cells to produce GLP-1, which stimulates insulin production, regulates glucose absorption, and sends satiety signals to the brain. 

### 3.2. K Cells

K cells in the proximal intestine secrete glucagon-like insulinotropic peptide (GIP) in the presence of glucose. GIP is released rapidly postprandially and leads to release of insulin as well as promotes lipid storage in adipocytes [30]. This process is also SGLT1-dependent but it is not currently known whether this process involves taste receptors.

### 3.3. Enterochromaffin Cells

Enterochromaffin (EC) cells are distributed throughout the GI tract; the EC cells secrete serotonin (5-HT) to mediate changes in motility and secretion as well as in transduction of visceral stimuli [31]. Sweet taste molecules have been reported in EC cells. Animal studies indicate intestinal EC cells express α-gustducin and T1R [32,33]. T1R3 and T2R have also been identified in human small intestinal EC cells which release 5-HT in response to stimulation with sucralose [34]. This suggests that one role for EC cells is nutrient sensing in the gut with subsequent release of 5-HT leading to a variety of downstream effects. 

## 4. Glucose-Sensing by Gut Endocrine Cells

Glucose in the intestinal lumen leads to the release of several regulatory peptides, including the incretin hormones GIP, GLP-1, and GLP-2 as well as 5-HT [35,36]. Several different mechanisms may be involved in glucose-sensing by gut enteroendocrine cells. Evidence suggests that glucose is metabolized, leading to generation of ATP and the closing of K_ATP_ channels in the cell membrane, a process that is similar to insulin release from the pancreatic β cell [37,38].

There is likely an additional mechanism of glucose-sensing in the gut, as GLP-1 is secreted in response to non-metabolizable sugars. As described above, glucose and non-metabolizable sugars are transported via SGLT-1. In addition to serving as glucose co-transporters, SGLT-1 and SGLT-3 may also be involved in glucose-sensing with subsequent release of 5-HT and GLP-1 [39,40,41]. 

Taste receptors in the gut may also be responsible for glucose-sensing. Elements of the sweet taste transduction pathway, including T1R2, T1R3, and α-gustducin are co-expressed in mouse and human enteroendocrine cells [24,32,42,43]. T1Rs may also play a role in the upregulation of SGLT-1 and GLUT2 in the intestinal epithelium in response to glucose as well as in the regulation of GLP-1 secretion [21,24].

## 5. Neuroanatomy of Sweet Taste

**Figure 3:**
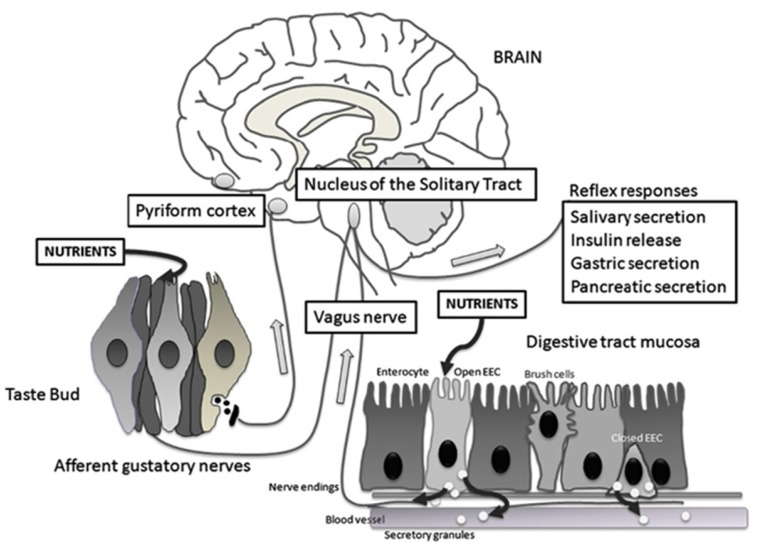
Schematic representation of the taste circuitry. The gustatory system is represented by taste cells in taste buds and their gustatory nerves. Corresponding to the gastrointestinal system, there are two enteroendocrine cells (EEC), one that is open to the lumen releasing cholecystokinin (CCK) and glucagon-like peptide 1 (GLP-1) in response to luminal nutrients and one that is closed. Vagal fibers are located underneath the GI mucosa in close contact with hormone secretions. The signals from the gustatory system reach the rostral nucleus of the solitary tract whereas visceral impulses terminate at the caudal nucleus of the solitary tract. From the nucleus of the solitary tract, gustatory and visceral information projects to several brain regions including the amygdala, the hypothalamus, and the ventral posterior nucleus of the thalamus. These regions are involved with ingestive motivation, physiological reflexes, and energy homeostasis. Reproduced with permission from [50].

Upon activation of sweet taste receptors, neural afferents of cranial nerves send gustatory information to the rostral division of the nucleus tractus solitarius (rNTS) of the medulla (Figure 3) [44]. In rodents, fibers then ascend ipsilaterally to the parabrachial nucleus (PBN) [45]. From the PBN, a dorsal pathway projects to the parvicellular part of the ventroposteromedial nucleus of the thalamus (VPMpc, the taste thalamic nucleus) and a ventral pathway to the amygdalar and lateral hypothalamic areas. Thalamic afferents then project to the primary gustatory cortex, which is defined as the VPMpc cortical target located within the insular cortex [46]. In primates and humans, rNTS projections bypass PBN and proceed directly to VPMpc [47].

Imaging studies have indicated that sweet, salty, bitter, and umami tastes activate distinct cortical fields in the mammalian primary gustatory cortex and suggest the existence of a gustotopic map in the brain [48]. A recent study demonstrated that direct activation of the cortical fields associated with sweet and bitter tastes elicits specific behavioral responses in mice using two different tasks [49]. In the first task, mice were placed into a two-chamber arena and a light stimulus was delivered to the relevant cortical field only when the animals entered a specific chamber. Mice expressing channelrhodopsin 2 (ChR2) in the sweet cortical field showed a preference for the chamber associated with light stimulation. Meanwhile, mice expressing ChR2 in the bitter cortical field demonstrated avoidance of that chamber. In the second task, mice were trained to lick from a water bottle on presentation of a cue. During licking, a light stimulus was applied to the relevant cortical field. Stimulation of the bitter cortical field in thirsty mice led to a marked reduction in licking. Conversely, light stimulation of the sweet cortical field led to an increase in licking. These findings demonstrate that activation of a specific taste cortical field can bring about specific behaviors that are characteristic of exposure to that taste.

## 6. Central Regulation of Food Intake and Energy Balance

Nutrient sensing initially occurs in the GI tract. Signals via vagal afferent neurons are sent directly to sympathetic neurons in hindbrain nuclei. These nuclei then project to forebrain areas such as the hypothalamus [51]. The gut thus sends signals to the rest of the body, including the brain, about current nutritional status by secreting hormones, such as ghrelin, GIP, PYY, CCK, and GLP-1, as well as neurotransmitters, such as 5-HT, that are important regulators of glucose and energy homeostasis [52].

These signals are then processed in different brain nuclei, including the melanocortin system. This system is composed of two peptide-expressing populations of neurons in the arcuate nucleus (ARC) and their downstream targets. One set of ARC neurons express the precursor peptide pro-opiomelanocortin (POMC), which is transformed into α-melanocyte-stimulating hormone and serves as an agonist for melanocortin receptor 4 (MC4R). Activation of POMC neurons results in anorexigenic effects, including decreased food intake and weight loss. The second set of neurons express neuropeptide Y (NPY) and the MC4R antagonist/inverse agonist agouti-related protein (AGRP). Activation of NPY-AGRP neurons results in increased food intake and weight gain [53,54]. Downstream sites for both neurons are located in the hypothalamus, including the paraventricular nucleus (PVN), ventromedial hypothalamus (VMH), and the lateral hypothalamic area [55].

**Figure 4:**
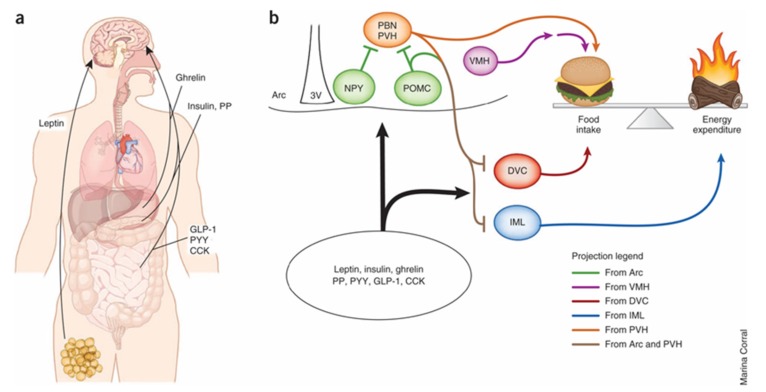
(**a**) Multiple peripheral factors have been shown to modify food intake and energy expenditure through direct effects on the central nervous system. Leptin, which likely acts on sweet taste receptor cells, is the primary regulator of energy balance and food intake in the hypothalamus. (**b**) Evidence suggests that melanocortin signaling regulates these physiological processes by means of distinct projection patterns originating from pro-opiomelanocortin (POMC) neurons in the arcuate nucleus (Arc). Sweet taste receptors likely act on neuropeptide Y (NPY) neurons in the hypothalamus to regulate energy homeostasis. Ultimately, MC4 receptor (MC4R)-expressing neurons downstream of POMC neurons act to suppress food intake and increase energy expenditure. Hypothalamic NPY/AgRP, paraventricular nucleus of the hypothalamus (PVH) and VMH neurons, as well as hindbrain dorsal vagal complex (DVC), parabrachial nucleus (PBN) and spinal cord intermediolateral cell column (IML) neurons, also regulate or counter-regulate these activities. PP, pancreatic polypeptide; PYY, peptide YY; 3V, third ventricle. Adapted with permission from [60].

The POMC and NPY-AGRP expressing neurons receive input from many different sources (Figure 4). Insulin and leptin may act as adiposity signals, as plasma levels are directly proportional to the amount of stored fuel in adipose tissue. Furthermore, insulin and leptin receptors are expressed throughout the hypothalamus, including on POMC and NPY-AGRP neurons in the ARC [56,57,58,59].

Sweet taste receptors also play a role in nutrient sensing in the hypothalamus similar to mechanisms used in the periphery. Neurons containing T1R2 and T1R3 have been identified in the CNS, including the hypothalamus. Recent data suggest that the majority of sweet taste receptor activity occurs on non-POMC leptin-responding neurons in the hypothalamus [61].

## 7. Central Actions of Gut Hormones

### 7.1. Effect of Leptin

Leptin is an anorexigenic hormone that is primarily produced by adipocytes. Leptin is the primary mediator in the hypothalamus that regulates energy balance and food intake [62]. Leptin acts on a specific obese receptor (Ob-R) which is encoded by the db gene [63]. Ob-R is expressed in several hypothalamic nuclei and leads to increased expression of POMC as well as simultaneous reduction of NPY-AGRP expression [64,65]. Mutations in leptin (*ob*/*ob*) or its receptor (*db*/*db*) produce mice that are hyperphagic and severely obese [66,67,68].

### 7.2. Leptin and Sweet Taste in Mice

Leptin displays a sweet suppressive effect in studies with mutant mice containing a point mutation of the *db* gene (*db*/*db* mice) that lack a functional leptin receptor (Ob-Rb) [69]. Chorda tympani nerve responses to various taste stimuli were compared in *db*/*db* and lean control mice before and after intraperitoneal (i.p.) injection of recombinant leptin. The chorda tympani (CT) nerve transmits taste information from the anterior two-thirds of the tongue [64]. The *db*/*db* mice demonstrated greater CT nerve responses to sweet compounds compared to lean control mice. However, after i.p. injection of leptin, CT nerve responses to sweet substances were significantly suppressed in control mice but not in *db*/*db* mice. Other substances, such as NaCl, HCl, and quinine were not affected by leptin administration, which suggests that leptin selectively affects sweet taste sensitivity. 

Leptin’s target appears to be on sweet taste receptor cells. Studies using real-time polymerase chain reaction (RT-PCR), in situ hybridization, and immunohistochemistry show that the functional leptin receptor Ob-Rb is expressed on taste bud cells, with approximately 30–40% of T1R3 expressing cells co-expressing Ob-Rb (unpublished data) [64]. Further studies show that application of leptin to isolated taste cells leads to a reduction of cell excitability [69]. This suggests that leptin acting on Ob-Rb may suppress sweet taste sensitivity by decreasing responsiveness of sweet taste cells.

### 7.3. Leptin and Sweet Taste in Humans

Plasma leptin levels show a diurnal variation in humans, with levels peaking around midnight and lowest around noon to mid-afternoon [70]. A study of 91 non-obese subjects demonstrated a link between plasma leptin levels and sweet taste sensitivity in humans [71]. Recognition thresholds for sweet, salty, sour, bitter, and umami tastes were measured using different concentrations of sucrose, glucose, saccharin Na, NaCl, citric acid, quinine HCl, and monosodium glutamate. The authors demonstrated that recognition thresholds for sweet substances were tightly linked with circulating leptin levels. This was not seen with other taste stimuli.

### 7.4. Effect of Endocannabinoids

Cannabinoids, such as *Cannabis sativa* (marijuana), have long been known to have an appetite-stimulating effect. However, endogenous endocannabinoids, including anandamide (AEA) and 2-arachidonoyl glycerol (2-AG), and their specific receptors, cannabinoid receptor type 1 (CB1) and cannabinoid receptor 2 (CB2), were only discovered in the 1980s [72,73]. CB1 receptors, which are located in the hypothalamus as well as peripherally, are likely involved in the orexigenic effects of endocannabinoids [74,75]. Evidence of these effects are demonstrated by injection of endocannabinoids in the hypothalamus which stimulates food intake while CB1 deletion in animal models leads to a lean phenotype and resistance to diet-induced obesity [76,77].

The endocannabinoid system is normally tonically inactive and only becomes transiently activated when needed. Leptin likely plays an important counter regulatory role. Genetically obese mice deficient in leptin (*ob*/*ob*) who were given a single i.p. injection of rimonabant, a CB1 receptor antagonist, showed reduced food intake [76]. Subsequent experiments where *ob*/*ob* mice were then treated with leptin demonstrated significant decreases in levels of endocannabinoids in the hypothalamus, but not in the cerebellum.

Recently, the relationship between endocannabinoids and leptin was further clarified. Jo et al. demonstrated that neurons containing melanin-concentrating hormone (MCH) in the hypothalamus project to the mesolimbic ventral tegmental area [78]. Thus, the area of the brain controlling appetite is linked to the region devoted to pleasure and reward. These MCH neurons are tonically inhibited by *γ*-aminobutyric acid (GABA) and receive input from the endocannabinoid system as well as leptin. When these MCH neurons are stimulated, it leads to an increase in intracellular calcium and release of endocannabinoids. This subsequently leads to the activation of CB1 receptors on GABA interneurons, which suppresses GABA release, increases excitability of MCH-containing neurons, and results in increased food intake. Conversely, when leptin receptors on MCH neurons are activated, voltage-gated calcium channels are blocked, suppressing endocannabinoid release, and this leads to an appetite-suppressing effect of leptin.

### 7.5. Sweet Enhancing Effect of Endocannabinoids

Endocannabinoids also likely enhance taste cell responses to sweeteners. Intraperitoneal administration of endocannabinoids led to a dose-dependent increase in CT glossopharyngeal nerve responses to sweeteners in mice [79]. This was not observed for salty, sour, bitter, or umami compounds or in CB1 knockout mice. These effects were also blocked by administration of AM251, a CB1 receptor antagonist, but not by AM630, a CB2 receptor antagonist. The authors further demonstrated by immunohistochemistry that sweet taste cells expressing T1R3 also express CB1 receptors. These findings suggest that endocannabinoids may enhance sweet taste response in sweet taste cells expressing T1R3.

## 8. Conclusions

Sweet taste receptors and sweet taste molecules are involved in transduction of sweet taste in taste buds. Furthermore, it is clear that sweet taste pathways are present in the gut and in the CNS, including the appetite center in the hypothalamus. Accumulating data suggest that these pathways act as nutrient sensors in the gut and the brain. They also serve to regulate energy balance, glucose homeostasis, and food intake. Interactions between peripheral and central pathways are carefully regulated with input from peripheral mediators, such as leptin, ghrelin, insulin, GLP-1, and endocannabinoids. Further elucidation of these pathways may provide invaluable insight into the pathogenesis of common diseases, including obesity and type 2 diabetes mellitus.

## Figures and Tables

**Table 1:** Enteroendocrine cells of the mammalian gastrointestinal tract. Adapted with permission from [15]. Several of the enteroendocrine cell types, notably A, K, and L cells, have subgroups or gradients along the intestine that contain different combinations of products; subgroups of I and L cells contain 5-HT.

Cell	Products	Luminal Receptors	Locations	Principal Effects
A (X-like) cells and subtypes	Ghrelin, nesfatin-1	T1R1-T1R3; T2Rs	Stomach	Appetite control, growth hormone release
Enterochromaffin cells *^,‡^	5-HT (5-HT is also contained in subgroups of I, K, and L cells)	FFARs 2, 3; TRPA1; toxin receptors; TLRs	Stomach, small and large intestine	Facilitation of intestinal motility reflexes and secretion; triggering of emesis and nausea in response to toxins
I cells	CCK (5-HT)	T2Rs; FFA1; GPR120; LPAR5; CaSR; TRPA1; TLRs	Proximal small intestine	Activation of gallbladder contraction and stimulation of pancreatic enzyme secretion
K cells, and subtypes	GIP	GPR119, GPR120; FFAR1	Proximal small intestine	Stimulation of insulin release
L cells, and subtypes ^‡^	GLP-1, GLP-2, PYY, oxyntomodulin (5-HT)	T2Rs; T1R2–T1R3; FFARs 1–3; GPR119, LPAR5, GPR120; CaSR	Distal small intestine, colon	Stimulation of carbohydrate uptake, slowing of intestinal transit, appetite regulation, insulin release
P cells	Leptin	Nutrient receptors	Stomach	Appetite regulation, reduction of food intake

* ECL cells do not contact the lumen. ^‡^ Sweet taste receptor molecules have been identified within L cells and enterochromaffin (EC) cells. Abbreviations: T1R, taste 1 receptor family; T2R, taste 2 receptor family; 5-HT, serotonin; ECL, enterochromaffin-like; FFAR, free fatty acid receptor; TRP, transient receptor potential; TLR, Toll-like receptor; FFA, free fatty acid; FFARs, free fatty acid receptors; GPR, G protein-coupled receptor; LPAR, lysophosphatidic acid receptor; CaSR, calcium-sensing receptor; CCK, cholecystokinin; GIP, gastric inhibitory polypeptide; GLP, glucagon-like peptide; PYY, peptide YY.
